# Circulating and tumor-based biomarkers predict clinical activity in cancer patients treated with the engineered anti-PD-L1 antibody MPDL3280A

**DOI:** 10.1186/2051-1426-2-S3-P136

**Published:** 2014-11-06

**Authors:** Marcin Kowanetz, Christina Rabe, Yuanyuan Xiao, Qun J Wu, Hartmut Koeppen, Cecilia Leddy, Rajesh D Patel, John Powderly, Holbrook Kohrt, Scott Gettinger, Jean-Charles Soria, Roy S Herbst, Rupal Desai, Mitchell Denker, Jane Ruppel, Marigold Boe, Rin Nakamura, Ling Fu, Teiko Sumiyoshi, Ahmad Mokatrin, Xiaodong Shen, Gregg Fine, Daniel S Chen, Priti S Hegde

**Affiliations:** 1 Genentech, Inc., USA; 2 Roche Diagnostics GmbH, Germany; 3 Carolina BioOncology Institute, NC, USA; 4 Stanford University, Stanford, CA, USA; 5 Yale University, New Haven, CT, USA; 6 Gustave Roussy, Villejuif, France; 7 Yale School of Medicine, New Haven, CT, USA

## 

PD-L1 expressed in the tumor microenvironment regulates Th1 immune responses and mediates cancer immune evasion through interactions with PD-1 or B7.1 receptors on activated T cells. MPDL3280A, an engineered human monoclonal antibody, targets PD-L1 and inhibits its function. To identify immunologic predictive and pharmacodynamic biomarkers of MPDL3280A treatment, we performed a comprehensive analysis of tumors and blood samples collected at baseline and/or on treatment from ≈280 patients with locally advanced or metastatic solid tumors, including NSCLC, RCC, melanoma and bladder cancer. Regardless of tumor type, clinical responses were characterized by PD-L1 expression, the presence of markers of T cell activation (Th1 gene signature and CTLA4), and the absence of fractalkine at baseline in the tumor microenvironment. Elevated baseline expression of IFN-γ and IFN-γ-inducible genes (e.g., *IDO1 *and *CXCL9*) was associated with MPDL3280A response in melanoma but not NSCLC or RCC. On treatment, responding tumors showed increased infiltration of Th1-dominant immune infiltrate and evidence of adaptive PD-L1 up-regulation. In contrast, progressing tumors displayed the following patterns of tumor-infiltrating lymphocytes (TILs) and PD-L1 expression: (1) few/no TILs and absent PD-L1 expression (immunologic ignorance), (2) TILs present with minimal/no PD-L1 expression (non-functional immune responses), or (3) TILs residing solely around the tumor cell mass outer edge (excluded infiltrate), suggesting that resistance to MPDL3280A may be associated with impaired T cell trafficking and/or function.

Profiling of ≈180 circulating biomarkers revealed that plasma concentrations of IL-18 and interferon-inducible T cell alpha chemoattractant (ITAC) increased in all patients following MPDL3280A treatment, representing a pharmacodynamic measurement of PD-L1 inhibition. In addition, analysis of PBMC showed an increase in T cell activation, as measured by IFN-γ and granzymes A and B gene expression in responders following MPDL3280A treatment, consistent with the observations in responding tumors. Baseline soluble PD-L1 was not associated with response. Some indication-specific biomarkers, such as plasma VEGF, decreased in responders with RCC but not with other indications. In NSCLC, a decrease in tumor burden markers, CA-125 and CEA, was associated with response. Similarly, IL-6 and IL-8 were differentially expressed on treatment in responders vs non-responders. Additionally, responders exhibited a decrease in circulating tumor DNA (ctDNA) in plasma, suggesting that ctDNA may be used to monitor MPDL3280A clinical activity in NSCLC.

In conclusion, these data provide general and indication-specific mechanistic insights into immune checkpoint inhibition, potential mechanisms of response and resistance, as well as identification of potential predictive and pharmacodynamic biomarkers of anti-PD-L1/PD-1 clinical activity across multiple tumor types.

